# Translating Fit and Strong!: Lessons Learned and Next Steps

**DOI:** 10.3389/fpubh.2014.00131

**Published:** 2015-04-27

**Authors:** Susan Hughes, Renae L. Smith-Ray, Amy Shah, Gail Huber

**Affiliations:** ^1^University of Illinois at Chicago School of Public Health, Chicago, IL, USA; ^2^University of Illinois at Chicago Center for Research on Health and Aging, Chicago, IL, USA; ^3^Northwestern University School of Physical Therapy, Chicago, IL, USA

**Keywords:** older adults, Fit and Strong, physical activity, longitudinal studies, arthritis

Fit and Strong! began in 1998. It grew out of the Hughes doctoral dissertation many years ago that examined the impact of a model long-term home care program for older adults. We learned at that time (1981) that arthritis was the most common chronic condition reported by homebound clients and the condition that was most frequently cited by them as interfering greatly with their function. To learn more about this story, we obtained funding from the National Institutes of Health (NIH) to conduct a prospective, longitudinal study in Chicago of 600 seniors who were unselected for presence of arthritis at baseline. We found again that arthritis was the most common condition reported and the number one cause of disability (1). We also measured participant joint impairment and conducted an analysis to try to determine which joints were causing the problem. Analyses clearly indicated that osteoarthritis (OA) in the lower extremity joints was the culprit, a scenario that makes sense when considering that people use these large weight bearing joints to perform most activities of daily living such as transferring, climbing stairs, and toileting (2).

We conducted the longitudinal study in order to understand the links between presence of OA and development of disability. Once we understood the causal chain, it was clear that our next step should be the development of an intervention to interrupt it. We examined the OA physical activity literature and found that people with OA have two problems. They are aerobically de-conditioned and have weaker muscles than age-matched controls (3, 4). People who have OA have a lot of pain in their joints. For those with lower extremity joint pain, the natural tendency is to stop moving around, which is, of course, the worst thing that people can do. A sedentary lifestyle leads to further joint stiffening, pain, muscle weakness, aerobic de-conditioning, and weight gain; potentially setting people up for the onset of co-morbid conditions like heart disease and diabetes (5–9). So we decided that our intervention must consist of a multiple component physical activity program that included aerobic walking and strength training. We also wanted to design a short term (8 weeks) program that had long-term results. Therefore, we talked to experts in the field and learned that we needed to also include a health education/behavior change component. Like the evidence-based Chronic Disease Self-Management Program (CDSMP), we borrowed heavily from the self-efficacy literature to design this piece that helps people gain mastery over their OA through an active lifestyle (10).

The resulting program, Fit and Strong!, consists of three 90-min sessions per week over 8 weeks. The first hour of each session is devoted to exercise (flexibility, aerobic, and lower extremity strengthening) and the last 30 min is devoted to a structured health education/group problem solving curriculum. We tested the program in an efficacy trial that found differential benefits in the treatment group on physical activity, self-efficacy for exercise, and lower extremity stiffness at 8 weeks. At 6 months, those gains were maintained and we saw additional benefits on self-efficacy for adherence to physical activity over time and lower extremity pain. Several of these gains were maintained at 12 months with large effect sizes (11).

## Translation Steps

### Change in instructors

The efficacy trial sought to demonstrate that a structured program of aerobic exercise and resistance training would not harm persons with painful lower extremity joints. The program was delivered by trained physical therapists who had experience working with persons with OA, but this was an expensive model for translation. By this time, we had obtained funding to test different ways of bolstering maintenance of physical activity after Fit and Strong! ends. This effectiveness trial was conducted on the south side of Chicago, enabling us to expand the reach of the program into largely African American communities. We used this study as an opportunity to conduct a natural experiment. We used the physical therapist instructor model with the first 200+ enrollees and then taught the remaining 300 enrollees using certified exercise instructors. Outcomes were very strong at 8 weeks and 6 months with both types of instructors, attendance was high and participant evaluations glowing (12). Therefore, we decided to move forward with the certified exercise instructor model. Overall long-term effects from this trial were very strong, including significant gains in physical activity over 18 months of follow up that were accompanied by improved lower extremity OA symptoms, observed performance gains in lower extremity strength, and mobility (risk factors for falls), and anxiety and depression out to 18 months (13).

### Partnership with AAA’s

We subsequently received funding from the Centers for Disease Control and Prevention (CDC) to test the translation of Fit and Strong! in partnership with Area Agencies in Aging in Illinois and North Carolina. This work with community partners enabled us to develop a license and fee structure, fine tune Lead (i.e., T), Master, and Instructor trainings, and develop many other types of materials including a program implementation guide and participant and instructor manuals. We also developed an interactive website that enables us to track participant attendance, conduct program evaluations, and collect a reduced set of outcome measures for all participants at baseline and immediately post-program. The outcomes now tracked across sites include: body mass index (BMI), lower extremity joint pain and stiffness, self-efficacy for exercise, engagement in physical activity, and energy/fatigue. We also learned along the way that some sites find it more practical to offer the program two times per week. We now allow providers to make this adaptation to the program when necessary as long as they cover the full complement of 24 sessions, which extends the program to 12 weeks in length.

### Hispanic version of Fit and Strong!

More recently, we developed and tested a new Hispanic version of the program, ¡en Forma y Fuerte!, in Chicago and Phoenix. That pilot taught us that many older Latinos who have immigrated to the U.S. have low levels of formal education. Our participant manuals are written for eighth grade literacy levels. We are now revising the Hispanic Manual to a fourth grade level and will work with instructors to use pictures and stories to get points across. We obtained participant baseline, 8-week and 6-month outcomes for this pilot. Preliminary analyses show strong results at both time points and we plan to publish the findings very soon (14).

### Lay leader effort

There is currently no process in place for providers to help graduates of an evidence-based program move on to a different, complementary evidence-based program. For this reason, we obtained foundation funding to examine initial steps that could be taken to bundle Fit and Strong! with other evidence-based programs like Matter of Balance and CDSMP. This lay leader effort is training people who have already been trained in an evidence-based program and layering the Fit and Strong! training on top. Currently, we are implementing the lay leader model in IL, TX, and MI, USA. Sites that are using the lay leader approach continue to use our interactive website to enter pre- and post-participant outcome assessments and attendance data. We will be analyzing the outcome data soon to learn whether participant outcomes with this new instructor model are as strong as outcomes seen with the physical therapist and certified exercise instructor models. Anecdotal feedback from participating sites has been very positive.

### Next steps

Finally, while offering the program on the south side of Chicago we were asked by participants to include more information in the Fit and Strong! participant manual about diet/weight management. We researched this issue, learned about the strong relationship between overweight/obesity and knee OA, and obtained funding from the National Institute on Aging to compare the effectiveness of customary Fit and Strong! to that of a new version, Fit and Strong! Plus, that includes both physical activity and an explicit dietary change/weight management component. The new program also has 24 sessions, but the health education curriculum has been adapted to include dietary behavior change information intended to facilitate participant weight loss. Early returns from this study have been quite positive (15). We are also currently working with a colleague in the Department of Psychiatry at University of Illinois at Chicago who is testing an adapted version of Fit and Strong! for persons who exhibit symptoms of depression. This pilot is currently underway with older veterans who have been seeking treatment for depressive symptoms. This effort to adapt Fit and Strong! for use with a specific clinical population is very similar to the effort reported in this issue in the Reynolds et al. article to adapt and test the program with cancer survivors (16). Similar to the Reynolds pilot, the depression pilot team also removed the arthritis-specific material from manual and replaced it with material on recognizing symptoms of depression and managing them with physical activity. We are very pleased to see that the pilot of the Reynolds et al., adaptation of Fit and Strong! for cancer survivors improved their engagement in physical activity, self-efficacy for aerobic exercise as well as symptoms of anxiety and depression.

To conclude, the enduring hallmark of an evidence-based program is the capacity to produce the same, consistent results across different populations, geographic sites, and instructors. Fit and Strong! has demonstrated the capacity to produce nearly identical participant outcomes across six different evaluations with Caucasian, African American, and Hispanic participants; across sites in IL, MI, NC, TX, and AZ; and with three different types of instructors – physical therapists, certified exercise instructors, and experienced evidence-based program lay leaders. Our program that combines structured physical activity with health education for building self-efficacy and behavior change is starting to demonstrate similar positive outcomes with additional clinical populations like cancer survivors and is being tested with persons with symptoms of depression. We are also very excited about the potential contribution of the new Fit and Strong! Plus program to not only promote a physically active lifestyle but also simultaneously promote healthy eating and weight management. As the foregoing demonstrates, the chapter is still very much being written on Fit and Strong! adaptations and outcomes, so stay tuned for future developments!

## Conflict of Interest Statement

The authors declare that the research was conducted in the absence of any commercial or financial relationships that could be construed as a potential conflict of interest.

This paper is included in the Research Topic, “Evidence-Based Programming for Older Adults.” This Research Topic received partial funding from multiple government and private organizations/agencies; however, the views, findings, and conclusions in these articles are those of the authors and do not necessarily represent the official position of these organizations/agencies. All papers published in the Research Topic received peer review from members of the Frontiers in Public Health (Public Health Education and Promotion section) panel of Review Editors. Because this Research Topic represents work closely associated with a nationwide evidence-based movement in the US, many of the authors and/or Review Editors may have worked together previously in some fashion. Review Editors were purposively selected based on their expertise with evaluation and/or evidence-based programming for older adults. Review Editors were independent of named authors on any given article published in this volume.
